# Tubular TPU/SF nanofibers covered with chitosan-based hydrogels as small-diameter vascular grafts with enhanced mechanical properties

**DOI:** 10.1038/s41598-022-10264-2

**Published:** 2022-04-13

**Authors:** Sasan Maleki, Amir Shamloo, Farnoosh Kalantarnia

**Affiliations:** 1 School of Mechanical Engineering, Sharif University of Technology, Tehran, Iran; 2 Stem Cell and Regenerative Medicine Center, Sharif University of Technology, Tehran, Iran

**Keywords:** Biological models, Biomedical engineering

## Abstract

Native grafts such as internal mammary artery and saphenous vein are the main choice for coronary artery bypass graft. However, due to the limitations associated with their availability and rapid failure caused by hyperplasia, small diameter tissue-engineered vascular grafts (TEVGs) with sufficient post-implantation patency are urgently demanded as artificial alternatives. In our previous work, we innovatively fabricated a bilayer vascular graft providing appropriate structural and biological properties using electrospinning and freeze-drying methods. It was proved that the mechanical properties of the proposed graft enhanced in comparison with using either of methods individually. Here, we adopted the same methods and incorporated an anticoagulant internal layer (inner diameter 4 mm), comprised of co-electrospun fibers of silk fibroin (SF) and heparinized thermoplastic polyurethane (TPU), and an external highly porous hydrogel fabricated by freeze-drying method. The electrospun layer exhibited strong mechanical properties including superior elastic modulus (4.92 ± 0.11 MPa), suture retention force (6.73 ± 0.83 N), elongation at break (196 ± 4%), and comparable burst pressure (1140 ± 12 mmHg) while the external hydrogel provided SMCs viability. The heparin was released in a sustain manner over 40 days, and the cytocompatibility and blood compatibility of scaffold were approved using MTT assay and platelet adhesion test. Thus, the proposed graft has a potential to be used as an artificial blood vessel scaffold for later in-vivo transplantation.

## Introduction

Nowadays, as a result of global aging, cardiovascular disease (CVD) is responsible for 17.3 million deaths annually, and the number is increasing to more than 23.6 by 2030^1^. Coronary artery diseases (CAD) are the major cause of CVD mortality throughout the world, which are mainly associated with atherosclerosis^2^. Based on clinical and anatomical characteristics, various approaches might be used as treatments of narrowing in coronary arteries, including percutaneous coronary intervention (PCI) and coronary artery bypass graft (CABG) surgery^3^. The CABG surgery is the main therapeutic option for high-risk patients with severe occlusion in the main artery branches^4^. Although using the internal mammary artery (90–95% 10-year patency) and saphenous vein (40–50% 10-year patency) is the first choice for CABG, adequate and suitable sources for autologous grafts are not always available^5^. Moreover, due to the sudden elevation of operating pressure, the smooth muscle cell (SMC) proliferation increases within the inner layer and results in intimal hyperplasia (limited to veins)^6^; hence, the need for creation of durable prosthetic arterial grafts is indisputable.

Large-diameter synthetic vascular grafts (> 6 mm) have shown acceptable results in aortic reconstruction (95% five-year patency) and less satisfactory results in infra-popliteal regions (< 50% one-year patency). CABGs may induce host responses, and deposition of platelets on the lumen, which lead to thrombosis and formation of a blood clot^7,8^. Small-diameter synthetic vascular grafts (< 6 mm) are not practical alternatives to CABG due to their rapid failure after the surgery which is associated with thrombosis in the grafted area^9^. In view of short-term patency rates of synthetic grafts and the possibility of rejection by the immune system, tissue engineering can be a promising solution to address the aforementioned limitations.

Tissue-engineered vascular grafts (TEVGs) are potential replacement vessels developed to mimic the properties of native arteries. Scaffolds can be fabricated using a wide variety of methods among which are phase separation, freeze-drying, freeze-gelation, and electrospinning^10,11^. The latter is a popular method to fabricate micro and nano-fibers from synthetic and natural materials^6^. Synthetic biomaterials can provide favorable mechanical properties while natural biomaterials possess suitable biocompatibility. A myriad of attempts has been made to meet different requirements of TEVGs. A common way is enhancing the structural and mechanical properties of TEVGFs by fabricating multilayer scaffolds that are able to mimic the layered structure of autografts. Thus, combinations of the aforementioned methods for fabricating multilayer grafts have been used by different researchers.

Another basic criterion for TEVGs is providing an anti-thrombotic lumen^12^. Endothelial cell seeding, improving endothelialization by adding vascular endothelial growth factor (VEGF)^13–16^, adding cell-adhesive peptides to the luminal surface, using antiplatelet drugs (e.g. aspirin)^17^, and employing anticoagulant drugs (e.g. heparin)^18^ have been reported as effective efforts to minimize the inflammatory reactions. Studies have revealed that heparin has a direct inhibitory effect on vascular SMCs proliferation^19–21^. Also, heparin can significantly diminish platelets aggregation after surgery^22^.

In the previous work, we successfully introduced a tubular heparinized vascular graft fabricated by co-electrospinning of Poly-caprolactone (PCL) and gelatin followed by freeze-drying the outer-layer gelatin hydrogel. Experiments result confirmed that the as-prepared scaffold had good cell and blood compatibility, and its mechanical properties were superior than those of each individual layer. Herein, we aimed to improve mechanical properties and the capacity of the scaffold in preventing platelet adhesion. TPU has been known for its blood compatibility as well as remarkable mechanical properties, and SF possesses low immunogenicity and excellent biocompatibility. The TPU/SF scaffold offers improved drug release profile, sufficient suture retention force, and adequate burst pressure for surgical procedure and enduring blood pressure. The small-pore inner layer provides a more suitable structure for endothelial cells while the outer layer supports SMCs proliferation. The applied approach resulted in enhancing both mechanical and hemocompatibility characteristics. Also, the morphology, release profile, and biocompatibility of the scaffold were assessed and compared with previous studies and native artery grafts. Also, the in-vitro cytocompatibility of scaffold was investigated on human umbilical vein endothelial cell line (HUVEC) and rat smooth muscle cell (a7r5 cell line).

## Materials and methods

### Materials

Bombyx mori silkworm cocoons were purchased from a local market. Chitosan (Medium molecular weight,), gelatin (type A, from porcine skin), dialysis cassette, and thiazolyl blue tetrazolium bromide (MTT color) were purchased from Sigma-Aldrich. Thermoplastic polyurethane (TPU, Mw = 85,000)) was purchased from Bayer, Germany. Heparin Sodium (5000 IU/ml with 200 unit/mg) was purchased from Caspian Tamin Pharmaceutical Company, Iran. Ethanol, polyethylene oxide (PEO, Mw = 900,000), lithium bromide (LiBr), sodium carbonate, tetrahydrofuran (THF), dimethylformamide (DMF), 1-ethyl-3-(3-dimethylaminopropyl) carbodiimide hydrochloride (EDC), N-Hydroxysuccinimide (NHS), toluidine blue, acetic acid, hydrochloric acid, sodium hydroxide and hexane were purchased from Merck. Rat smooth muscle cell (a7r5) and human umbilical vein endothelial cell line (HUVEC) were obtained from Pasteur Institute, Tehran, Iran. Fetal bovine serum (FBS), DMEM, DMEM/F12 (1/1), 1% penicillin/streptomycin, Trypsin/EDTA, and DMSO were purchased from BioIdea, Iran.

### Preparation of aqueous SF solution

The silk fibroin (SF) solution was prepared as previously described^23^. Briefly, the cocoons were cut into small pieces and boiled in 0.02 M sodium carbonate aqueous solution for 30 min, and then rinsed with distilled water. Then, the extracted SF was dissolved in 9.3 M LiBr solution at 60 °C for 4 h. The obtained solution was dialyzed against distilled water, using a dialysis cassette (3500 MWCO), for 49 h. The final concentration of SF was determined by weighing the solid mass of 1 ml of the solution after drying. The final concentration of SF was approximately 7% (*w*/*v*).

### Electrospinning of inner layer

TPU nanofibers were fabricated by electrospinning of 5 ml of 8, 10 and 12 wt% polymer dissolved at DMF/THF solution (1/3 v/v). The TPU solutions were delivered at a constant rate of 0.5 ml/h. The distance and the voltage between collector and needle were 18 cm, and 18 kV respectively. In order to fabricate silk fibroin nanofibers a solution of 1.5 wt% PEO were prepared and stirred on a magnetic stirrer for 24 h, and added to SF solution to obtain PEO/SF solution (1/3 v/v). The distance and the voltage between collector and needle were 22 cm, and 14.5 kV, respectively, and the flow rate of the solution was 1.2 ml/h. All the solutions were delivered by standard 5-ml syringes attached to 18 G stainless steel needles. The fibers were collected on a grounded aluminum rod foil with the diameter of 4.5 mm, which was rotating at the 1000 rpm speed.

Moreover, another sample was fabricated by co-electrospinning of TPU (10 wt%) and SF. The electrospinning parameters for this sample were as the same as previously described parameters. Electrospinning parameters of different electrospun scaffolds are presented in Table 1.

**Table 1:** Electrospinning parameters.

	Polymer solution					Electrospinning parameters					
	Polymer solvent		Polymer concentration	Span80 concentration (μl)		Heparin (μl)		Flow rate (ml/h)	Distance (cm)	Voltage (kV)	collector speed (rpm)
TPU	DMF/THF 1/3(*v*/*v*)		8, 10, 12 (*w*/*v*)	–		–		0.5	18	18	1000
SF	PEO (1.5%*w*/*v*)		PEO/SF 1/3(*v*/*v*)	–		–		1.2	22	14.5	1000
TPU-Hep	DMF/THF 1/3(*v*/*v*)		10(*w*/*v*)	25		250		0.5	18	18	1000

### Outer-layer fabrication

**Figure 1:**
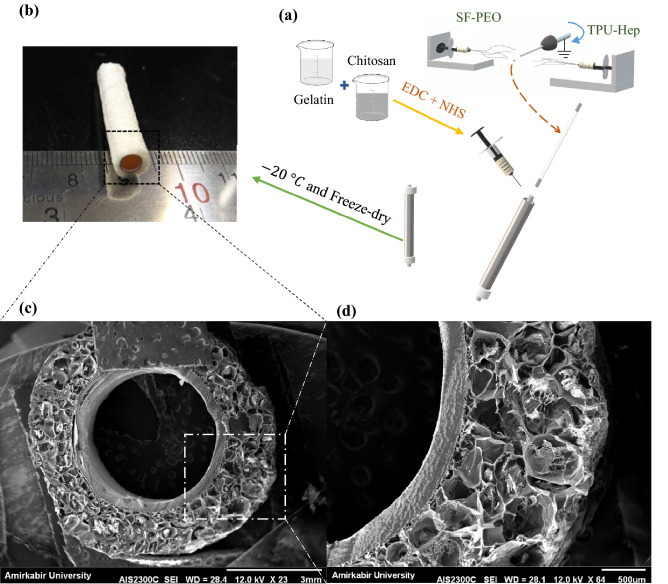
(**a**) Scheme of bilayer heparinized vascular scaffold combining electrospinning and hydrogel fabrication. (**b**) Macrostructure of the scaffold. (**c**, **d**) SEM images of the cross-sectional portion of the scaffold. Scale bars as indicated: (**c**) = 3 mm, (**d**) = 500 μm.

SMCs proliferation requires interconnected porous scaffold with pores larger than 60 μm. Therefore, the freeze-drying method was used to create 3D scaffolds with large pores and suitable for large cells. In order to fabricate the outer-layer hydrogel, gelatin (5 wt%) was dissolved in distilled water, and chitosan (3 wt%) was dissolved in distilled water containing acetic acid (2% *v*/*v*). Different proportions of chitosan/gelatin solutions were subsequently mixed (1/1, 2/1, and 3/1 v/v -abbreviated as C/G 1/1, C/G 2/1, C/G 3/1 samples, respectively). EDC and NHS were used as cross-linkers, and their final concentrations in aqua solution were adjusted to 0.3 wt%. After fabricating the inner-layer by electrospinning method, the collector was placed coaxially in a tube with the inner diameter of 6 mm, the blend solution was then cast in the space between the collector and tube and was placed at – 20 °C. After 24 h, samples were immersed in 1% NaOH solution for an hour and washed three times. Afterward, the bilayer scaffolds were placed at – 20 °C overnight and then freeze-dried for 24 h (Fig. 1a). Figure 1b shows the macroscopic appearance of the bilayer TEVG, and Table 2 summarizes the extracted data from SEM images.

**Table 2:** Extracted data from SEM images.

	Fiber diameter (μm)	Pore diameter (μm)		Porosity (percentage)
TPU8	0.6765 ± 0.29	–		–
TPU10	0.6963 ± 0.28	–		–
TPU12	0.9212 ± 0.38	–		–
SF	1.0273 ± 0.36	–		–
TPU-Hep	0.8130 ± 0.39	–		–
Freeze-dried C/G 1/1	–	285.60 ± 102.51		63.6
Freeze-dried C/G 2/1	–	320.57 ± 132.05		68.55
Freeze-dried C/G 3/1	–	410.65 ± 177.15		61.32

### Loading of heparin

Heparin sodium solution was encapsulated into the core of TPU core–shell nanofibers via emulsion electrospinning. First, 5 ml of 10 wt% TPU solution was prepared as previously described. Next, 25 μl of Span 80 was added to the solution. After 2 h, 250 μl of injectable heparin sodium solution (5000 IU/ml) was added dropwise to the solution. Then, the obtained emulsion was stirred overnight to achieve a uniform electrospinning solution. Electrospinning of heparin-loaded emulsion was done under the conditions described in Table 1.

### Scanning electron microscopy (SEM)

The morphology and microstructure of scaffolds were examined using scanning electron microscopy (SEM, AIS2100, Seron Technology). The cross-section of samples was cut using a sharp blade and sputter coated with gold. The average fiber diameter and the hydrogels pore size were measured through analyzing the SEM images using an image processing software (ImageJ®). At least 50 segments of fibers and 50 pores were chosen randomly for each sample.

### The outer layer hydrogel characterization

#### Biodegradation behavior

To simulate *in-vivo* conditions, the weight loss assay in 5 ml PBS solution was used to determine the degradation rate of the samples. Samples of C/G were prepare, and their initial weight was measured before the incubation at 37 °C. PBS solution was replaced with fresh PBS every 48 h. Samples were taken out at time intervals and rinsed with distilled water to remove the remaining salts. Then, samples were freeze-dried, and the dry weight of samples were measured (*n* = 3). Degradation percentage was calculated using the following formulation:

1 $$ \% Degradation = \frac{{W_{0} - W_{i} }}{{W_{0} }} \times 100 $$

In this equation, *W*_0_ is the initial weight and *W*_*i*_ is the weight of dried sample at the time interval.

#### Swelling ratio

To determine the swelling ratio, freeze-dried hydrogels were weighed initially (*W*_*i*_) and immersed in distilled water. Then, swollen hydrogels were removed from distilled water at different time intervals and dried superficially with filter papers and weighed subsequently (*W*_0_). The swelling ratio was calculated using the following equation (*n* = 3):

2 $$ \% Swelling = \frac{{W_{0} - W_{i} }}{{W_{0} }} \times 100 $$

### Toluidine blue assay

A colorimetric method based on toluidine blue assay was used to determine the release profile of heparin over time^24–26^. First, toluidine blue solution was prepared by dissolving 5 mg of it in the 100 ml aqua solution (0.01 M HCL and 0.2 wt% NaCl). Afterward, different volumes of the heparin solution containing known amounts of heparin were diluted to two milliliters and added to three milliliters of toluidine blue solutions. Then, the mixtures were incubated at 37 °C. After one hour, two milliliters of n-hexane were added to each mixture, and the reacted dye was extracted into the organic layer by well-shaking the mixture for several minutes. Then, three milliliters of the blue solution under the organic layer were taken out and analyzed by UV spectrophotometer (Rey Leigh UV-2601) at 632 nm.

### Release profile of heparin

Three samples of heparinized scaffold and a drug-free specimen with almost the same weight were prepared and soaked in 5 ml PBS and then incubated at 37 °C and 120 rpm. At time intervals, 2 ml of the PBS solution was taken out and analyzed using the colorimetric method described in Sect. 2.8 and then replaced with equal amount of fresh PBS. Cumulative release of heparin (*M*_*i*_) was calculated using the following formulation:

3 $$ M_{i} = C\left( i \right)V + \sum C\left( {i - 1} \right)V_{s } $$

where *C*(*i*) is the concentration of heparin at time *i*, *V* is the total volume of release medium, and *V*_*s*_ is the volume of sample used for colorimetric method.

### Mechanical properties

#### Longitudinal tensile property

Longitudinal Tensile property were investigated using a unidirectional tensile machine (Hounsfield H10KS, UK) equipped with a 500 N load cell. Samples were cut into 2 cm × 1 cm pieces and soaked in PBS for 2 h The longitudinal mechanical before testing. Then, the sample was fixed between two clamps and stretched at a rate of 2 mm/min. Young modulus was calculated as the slope of the elastic region of the stress–strain curve, and the average amount of three samples was reported for each scaffold.

#### Burst pressure

Increasing the internal pressure was used as the method of measuring the burst pressure. Shortly, one end of the sample was closed, and liquid Vaseline was injected from the other end with a syringe pump at 4 mm/min. The maximum pressure before rupture was recorded by a pressure gauge (WIKA, Germany) and reported as the burst pressure.

#### Suture retention force

Suture retention force was measured using a unidirectional tensile machine. The samples were soaked in PBS for 2 h before testing. Then, one end of the sample was fixed with the lower clamp, and a 5–0 monofilament suture (Ethicon, Inc., Somerville, NJ) was passed through the other end at 2 mm from the edge. The suture was connected to the other clamp and pulled at a rate of 2 mm/min. The average of maximum force before pull-through of suture was reported as the suture retention force (*n* = 3).

### Water contact angle

The water contact angle test was conducted as an indication of the wettability of the electrospun layer. To measure static contact angle, the sessile drop method with distilled water was used. The values were obtained by dropping 4 μl of liquid on different sites of the samples. At least 3 separate drops were examined for each sample, and average measured values per sample were reported as the water contact angle.

### Biocompatibility

#### *In-vitro* cell culture

HUVECs were cultured in DMEM/F12 (1/1/*v*/*v*) medium supplemented with 10% FBS, and a7r5 cells were cultured in DMEM medium containing 10% FBS. The culture flasks were placed in a 5% CO_2_ incubator at 37 °C, and the medium was replaced every 48 h.

#### MTT assay

Cytotoxicity of scaffolds was investigated using MTT assay as an indirect indicator. Three samples of each electrospun scaffold and hydrogels were cut into small pieces and placed in 24-well plates. Samples were sterilized by immersing in 70% ethanol for two hours followed by three washes in sterile PBS for 20 min to extract the remained ethanol and then exposing to UV light for 20 min per each side. Next, the sterilized samples were soaked in the culture medium overnight.

The fifth-passaged HUVECs were cultured on electrospun samples at a cell density of 45,000. The a7r5 cells with the density of 50,000 at passage 6 were seeded in hydrogels and cultured in a 5% CO_2_ incubator at 37 °C, and the culture medium was replaced each 48 h. At 1, 3, and 7 days after cell seeding, the culture medium was removed. A 5 mg/mlsolution of MTT color was dissolved in PBS and sterilized by filtering. Then, 50 μl of the solution and 450 μl of fresh culture medium were added to each well, and the plate was incubated for 4 h. Afterward, the medium was removed. and 500 μl of DMSO was added to each well, and the plate was shaken vigorously for 15 min. The absorbance of the obtained solution was measured using a microplate reader (MPR4 +) at 570 nm.

#### Cell adhesion

To validate cytotoxicity results, cells were fixed on scaffolds and examined using SEM. Sterilized scaffolds were loaded with 50,000 cells at the passage 6. Cells were fixed on scaffolds by using the ensuing procedure: first, the medium was removed, and the samples were washed by PBS to dispose of unattached cells. Afterwa, glutaraldehyde 2.5% was added to each well. After 4 h, glutaraldehyde was removed, and dehydration was carried out by immersing scaffolds in a gradient of ethanol/distilled water solutions (50, 60, 70, 80, 90, and 100%). Finally, samples were allowed to dry overnight at 4 °C.

#### Platelet adhesion

PRP with the concentration of 10^9^ platelets/ml was purchased from Blood Transfusion Center of Tehran and diluted to the concentration of 10^8^ platelets/ml. Sterilized scaffolds were placed in a plate, and 200 μl of diluted PRP was added to each well, and the plate incubated at 37 °C. After 4 h, scaffolds were rinsed three times with PBS to remove unattached and loosely adherent platelets. Next, adhered platelets were fixed wi glutaraldehyde 2.5%, and samples were dehydrated by gradient ethanol solutions and examined by SEM.

## Results and discussion

### Morphology

**Figure 2:**
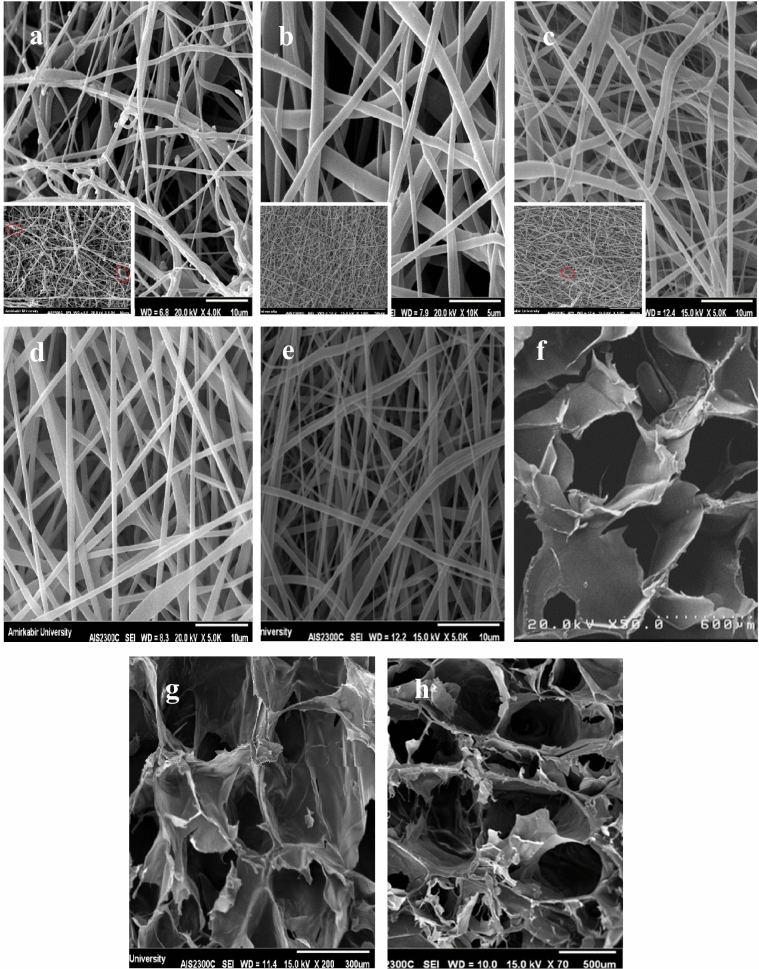
SEM images of (**a**) TPU8 (red ovals indicate beads), (**b**)TPU10, (**c**)TPU12, (**d**) SF, (**e**) TPU10-Hep, (**f**) C/G 1/1, (**g**) C/G-2/1, (**h**) C/G-3/1.

The SEM images of cross-sectional portion of bilayer scaffold is illustrated in Figs. 1c,d, and 2 contains examples of the microstructure of nanofibers and porous hydrogels. Also, the summary of average dimensions has been exhibited in Table 2. The thickness of the electrospun layer was about 0.3 mm, showing a good agreement with the reported thickness for tunica intima (100–400 μm)^27^.

Different electrospinning parameters (e.g., solution viscosity, conductivity, and surface tension) alter nanofibers' morphology and uniformity, which have profound effects on the biological and biomechanical properties^28,29^. In this study, all the electrospinning parameters such as applied voltage, flow rate, and collecting distance were kept constant, but the polymer concentration increased. Electrospun samples possessed fiber diameters ranging from 675 to 921 nm and showed a tendency to increase the fiber diameter as the polymer concentration increased, as reported in other studies^30^. The TPU12 (Fig. 2c) had the most concentration and viscosity among TPU solutions, resulting in thicker fibers. The electrospun TPU10 sample (Fig. 2b) was comprised of homogeneous and randomly distributed fibers with smooth surfaces, while the TPU8 sample (Fig. 2a) had a high density of beads and discontinuity in nanofibers morphology. Moreover, the dufferences in diameter might have been caused by the differences in the conductivity of solutions; in a way that as the conductivity increases, fibers elongate, and much thinner fibers will be generated^31^. Also, adding ionic salts, surfactants, polyelectrolytes, and drugs changes the solution's conductivity and charge density^21,31^. As shown in Fig. 2e, incorporation of heparin and Span80 into TPU10 solution resulted in the increase of fiber diameter, 17% thicker than TPU10 fibers. The average diameter of the SF sample (Fig. 2d) was approximately 1 μm.

The blood vessels consist of three layers which are different in structure and mainly composed of ECs, SMCs, and fibroblasts. A real challenge of producing TEVGs is to develop a proper porous structure to facilitate cellular interactions. Jue Hu, et al. have reported that SMCs failed to penetrate into the nanofiber scaffolds with an average fiber diameter of less than 1 µm^32^. Also, other studies have suggested that a too-small pore size limits pore interconnection and sufficient cell–cell interactions while a too-large pore size results in lower cell density^33,34^. In this study, freeze-dried hydrogels (Fig. 2f-h) showed a uniform porous structure and pore size of approximately 285–410 μm and an average thickness of approximately 1.5 mm. Although the thicker graft increases the surgery time, a certain thickness can ensure the mechanical properties; So that the increased wall thickness is associated with the robustness of the graft^35,36^. The suitable pore size and high porosity of hydrogels provide favorable conditions for cell proliferation and cell adhesion. Moreover, fiber diameter limits the inner-layer porosity and prevents SMCs infiltration into the inner layer.

### The outer layer hydrogel characterization

#### Biodegradation behavior

**Figure 3:**
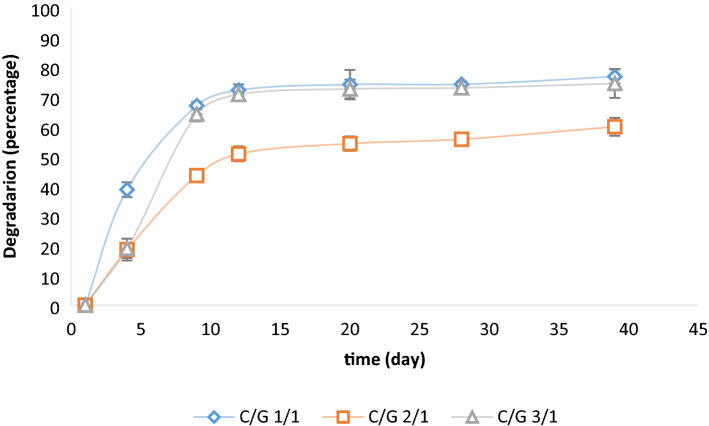
*In-vitro* degradation of hydrogels.

Lin et al. investigated the effect of the hydrogel properties on SMCs proliferation and suggested that a fast degradation rate is required to create sufficient space for cell spreading within the hydrogel^37^. Adelow et al. showed that the SMCs degraded the hydrogel to spread, migrate, and establish 3D cell–cell interactions^38^. However, a high degradation rate can compromise other scaffold's properties (e.g., integrity, stiffness, and cell adhesion). Thus, the overall performance of the hydrogel must be observed, and the polymer concentrations must be adjusted to optimize the hydrogel properties. We monitored the degradation rate of outer layer hydrogels as a function of incubation time in PBS at 37 °C which showed a good agreement with previous studies^39^. As Fig. 3 shows, by immersing hydrogels in PBS, the scaffolds immediately started to decompose, and by increasing the incubation time, degradation continued. Also, all hydrogels showed a similar pattern, losing a significant percentage of their weights during the first days and keeping a smoother weight loss until the end of the experiment. From the figure, it can be seen that the ratio of C/G had a significant role in weight loss. The samples with the ratio of 1/1 and 3/1 showed a significant mass loss while C/G 2/1 hydrogel had the least degradation rate. The 3/1 and 1/1 C/G hydrogels demonstrated about 64% weight loss during the first 10 days, and the rate of degradation after 10th days decreased. Similarly, C/G 2/1 degraded up to 43% of its weight in the first 10 days but relatively slowly during the next 30 days which is long enough for SMCs to proliferate through the hydrogel.

#### Swelling ratio

**Figure 4:**
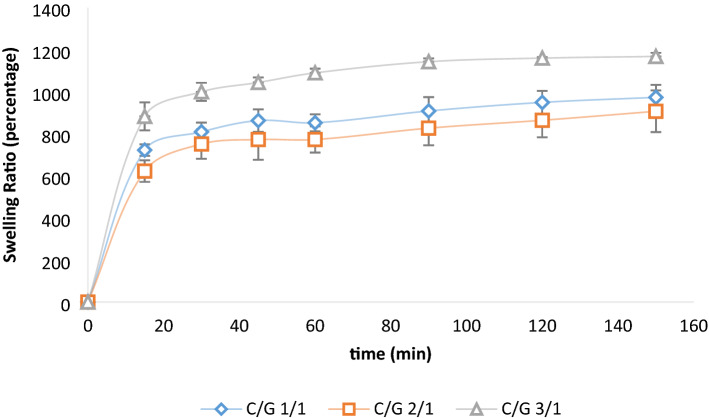
Swelling ratio of hydrogels.

The swelling ratio of hydrogels plays a significant role in determining the cell behaviors^40^. It has been reported that SMCs proliferate more quickly on stiff substrates and migrate toward the less hydrated surface becuase it is stiff enough for cell adhesion^41,42^. Figure 4 shows that swelling percentages for all hydrogels increase rapidly during the first 15 min and then rising to the equilibrium in 150 min. Studies reprted that chitosan can strongly affect the water content and increase it^39,43^. As the figure demonstrates, C/G 3/1 was the most swollen gel, which could be due to the strong polymer–polymer interactions and hydrophilicity of structure provided by dense hydrogen bonding in C/G 3/1 sample. Also, C/G 2/1 was the sample with the lowest swelling ratio. Owing to the biodegradation results, it can be concluded that C/G 2/1 had the highest crosslinking percentage. Thus, more hydrophilic groups (i.e. OH, NH_2_, and COOH) have been consumed by crosslinking process that caused a decline in the hydrophilicity of the sample^43^. Considering the biodegradation and swelling ratio, the C/G 2/1 sample possessed the most suitable degradation and swelling behaviors among the hydrogel samples.

### Release profile of heparin

**Figure 5:**
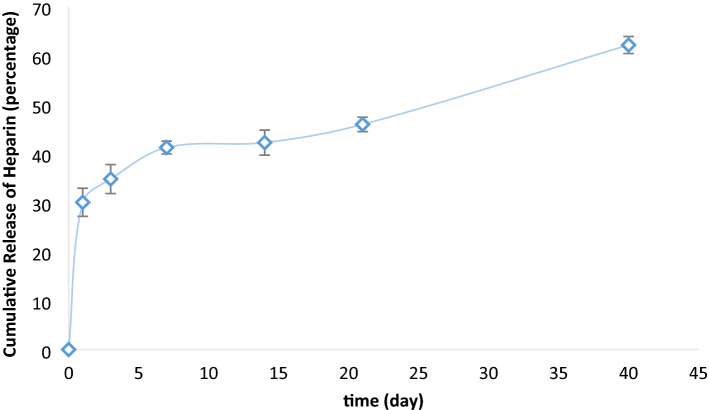
Release profile of heparin from TPU10 fibers.

Figure 5 represents the heparin release from TPU10 fibers. Studies have shown that depending on the severity of the injury, SMCs proliferation reaches its maximum during the first week after injury and can continue at high levels for 14–28 days^44^. Heparin treatment has shown effective results in preventing SMCs proliferation into the surface of grafted vessels, but due to the short half-life of heparin in the body, sustained delivery of heparin is required. The heparin release study was carried out on electrospun TPU (10 wt%) fibers over 40 days, and a total 62% of heparin was released at the end of the experiment, providing an adequate dose of heparin over this period. Compared with our previous work, TPU fibers showed more extended-release than PCL fibers (the total amount of loaded heparin released over 30 days) and gelatin fibers (98% over 14 days)^8^. According to Fig. 5, TPU-Hep had burst release of heparin at the first days, and almost 34 percent of heparin had been released within the first 3 days. Clowes et.al showed that heparin administration only at the first 3 days after injury was much more effective in preventing SMCs accumulation than postponed administration^45^. Therefore, the immediate release of heparin within the first days may be beneficial in preventing vessel failure by formation of blood clot or hyperplasia.

### Mechanical properties

An ideal TEVG should possess similar properties to the native artery and get integrated into it since any mismatch between mechanical properties of the TEVG and the adjacent blood vessels can cause a rupture in the graft or blood leakage. The mechanical properties of electrospun fibers are highly dependent on raw materials. Also, Johnson et al. illustrated that the burst pressure of the electrospun vascular grafts, fabricated from different polymers, increased as the thickness increased^46^. In this section, the results of the mechanical evaluation of scaffolds have been discussed and compared with those of the native grafts. The uniaxial tensile properties, burst pressures, and suture retention forces for different inner layers and native grafts are presented in Table 3.

**Table 3:** Summary of mechanical properties of fabricated samples and some native tissues.

	Tensile testing			Suture retention force (N)	Burst pressure (mmHg)
	Young modulus (MPa)	Tensile strength (MPa)	Elongation at break (%)		
SF	1292 ± 0.40	6.30 ± 0.51	5 ± 0.72	–	575 ± 17
TPU10	3.62 ± 0.15	–	–	–	1338 ± 43
TPU-SF	15.08 ± 0.45	5.54 ± 0.27	563 ± 36	10.05 ± 1.36	1267 ± 31
TPU-SF-Hep	4.92 ± 0.11	19.68 ± 0.94	196 ± 4	6.73 ± 0.83	1140 ± 12
Coronary arteries^53,54^	1.41 ± 0.72	–	–	1.96 ± 1.1	–
Saphenous vein^54,55^	23.7	6.3	17, 83	1.92 ± 0.02	1600–2500
Left internal mammary^56,57^	16.8	4.3	59	1.49 ± 0.49	2000

#### Longitudinal tensile property

Young's modulus for SF, TPU10, TPU-SF, and TPU-SF-Hep were 136.49 ± 0.40, 3.62 ± 0.15, 15.08 ± 0.45, and 4.92 ± 0.11 MPa, respectively. As reported, SF is a stiff biomaterial^23,47^; our results showed that SF fibers had a high elastic modulus with a low elongation at break (5 ± 0.72). On the other hand, electrospun TPU10 fibers showed a lower elastic modulus and a higher strain before detaching from the clamps. Young's modulus can be used to evaluate the capacity of materials for resisting deformation; so, as the Young's modulus increases, the compliance decreases. Co-electrospinning of SF and TPU10 offered the ability to fine-tune the mechanical characteristics of resultant nanofibers. As reported in Table 3, when TPU10 fibers and SF fibers were combined, the elastic modulus was almost five times higher than the pure TPU10 sample, and the sample elongated to 563 ± 36%. Moreover, the flexibility of electrospun polyurethane graft can be attributed to other mechanical properties such as tensile strength and elongation of break^48^. Adding heparin to fibers by emulsion electrospinning of TPU-SF-Hep led to decreased elastic modulus and elongation at break; however, comparing the Young's modulus, tensile strength, and elongation at break for the TPU-SF-Hep with those reported for the native grafts reveals that our electrospun layer exhibited sufficient strength and flexibility for vascular transplantation.

#### Burst pressure

The ability to endure the load from pressurized blood flow without a rupture or dilation is one of the main prerequisites of a functional TEVG. Due to the variation in blood pressure, a burst pressure of about 260 kPa is necessary for an artificial graft^49,50^. The saphenous vein is one of the widely-used autologous grafts and has a burst strength of about 1600–2500 mmHg. Electrospun fibers of TPU and co-electrospun fibers of TPU-SF and TPU-SF-Hep had the burst pressures of 1338 ± 43, 1267 ± 31, and 1140 ± 12 mmHg, respectively. Although the values for the abovementioned electrospun layers were less than native grafts, the burst strength of the TPU-SF specimen was in an acceptable range compared with autologous grafts and offered higher burst strength than our PCL/Gelatin graft (882 ± 56 mmH)^8^. Moreover, normal blood pressure ranges from 80 to 140 mmHg, which is much less than the pressure resisted by our scaffolds. Also, the burst strength can be further improved when the outer layer is fabricated, and scaffolds are cellularized.

#### Suture retention force

Sufficient suture retention strength can facilitate manipulation of grafts during the vascular transplantation. The suture retention forces for TPU-SF and TPU-SF-Hep were 10.05 ± 1.36 and 6.73 ± 0.83 N, repectively, which were significantly superior than those of the native tissues. Although heparin encapsulation into the core of TPU core–shell fibers reduced the suture retention strength, both heparinized and non-heparinized TPU-SF fibers possessed more strength than the generally accepted suture retention strength (> 2 N) for implantation^51^.

### Water contact angle

**Figure 6:**
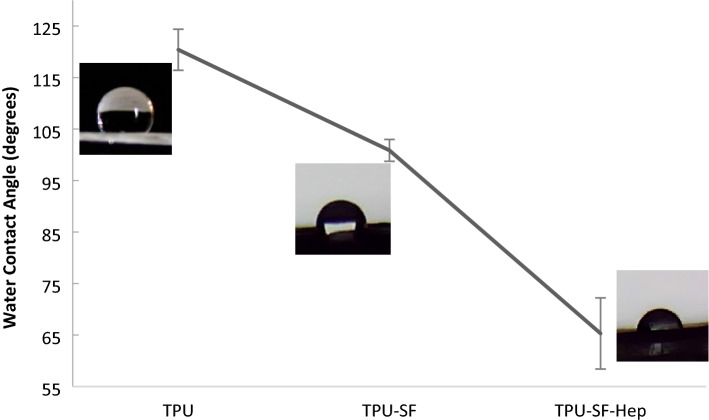
Water contact angle of different electrospun fibers.

Hydrophobicity of the surface plays a critical role in cell compatibility, attachment, and proliferation^52^. The TPU10 sample was hydrophobic (Fig. 6), and the contact angle of the sample was estimated to be 120.4° ± 3.98°, which was decreased to 100.86° ± 2.11° by co-electrospinning of SF fibers. Heparin has a high charge density and high hydrophilic capacity. Thus, after heparin were incorporated into the TPU-SF, the contact angle dramatically reduced to 65.31° ± 6.9°, suggestive of the enhanced hydrophilicity obtained by the presence of heparin in the construct. In the case of TPU10 fibers, the contact angle did not change over 15 s of testing while the contact angle of the electrospun TPU-SF and TPU-SF-Hep fibers reduced to zero by increasing the time to five and three seconds, respectively.

### Biocompatibility

#### MTT assay

**Figure 7:**
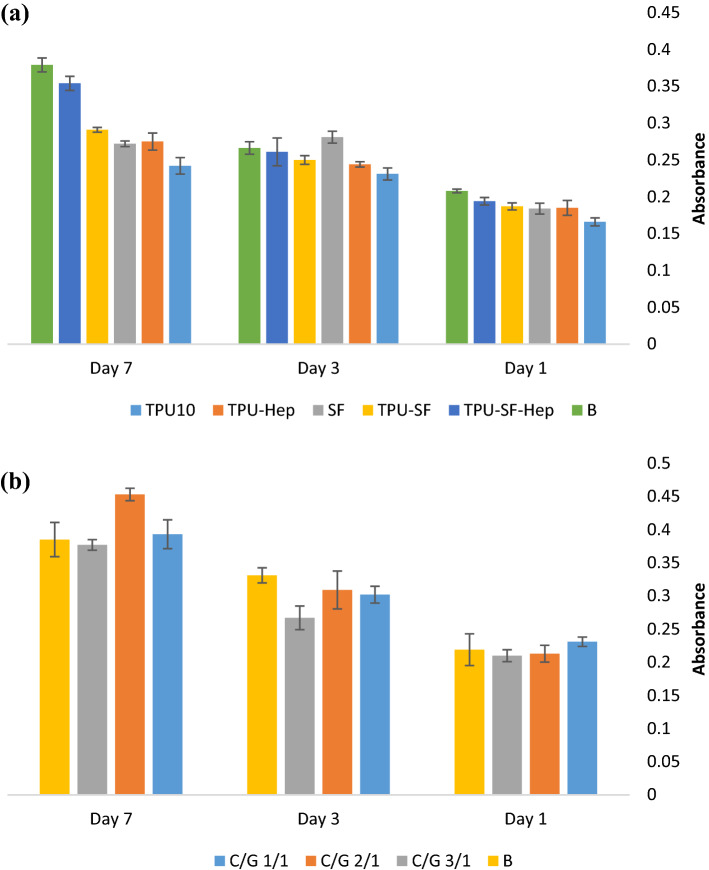
MTT cell viability assay (**a**) HUVECs on internal electrospun layer and (**b**) SMCs on external hydrogel layer.

MTT assay was performed on electrospun samples of TPU10, SF, TPU-Hep, TPU-SF, TPU-SF-Hep, and freeze-dried hydrogels to evaluate cytotoxicity of the scaffolds at 1, 3, and 7 culture time point. The results collected on day 1 showed that all electrospun samples proliferated in the same intensity (Fig. 7a). The MTT assay indicated that during the test, the TPU10 samples exhibited the lowest optical density (OD) compared with other samples, and consequently, cell growth on these fibers is less than other samples. Low cell adhesion and proliferation on TPU fibers is attributed to the hydrophobicity of the surface. The heparin administration increased wettability of the surface and promoted better adherence of HUVECs compared with the pure TPU10 samples. SF fibers had better cell compatibility and the OD for SF samples increased on the third day of incubation time, but decreased over one week which is because of the elevated cell coverage leading to cell death. Incorporating the SF to the TPU10 and TPU-Hep fibers resulted in fabricating multiscale fibers in the structure and induced higher cell growth on co-electrospun fibers of TPU-SF and TPU-SF-Hep.

Cytotoxicity of outer-layer hydrogels was evaluated using SMCs (Fig. 7b) at 1, 3 and 7 days after cell seeding. All C/G (1/1/, 2/1, and 3/1 v/v) hydrogels presented no cytotoxicity and showed almost same cell growth compared with the blank sample, but the C/G 2/1 hydrogel had the most OD, even greater than the blank sample, over one week. In our previous study, we used low concentration of glutaraldehyde (GA) as a chemical crosslinker for gelatin hydrogel. However, the cell proliferation was affected by the presence of GA in the structure. In the present work, a combination of EDC and NHS was used which resulted in better cell compatibility in comparison with gelatin hydrogel crosslinked by GA.

#### Cell adhesion

**Figure 8:**
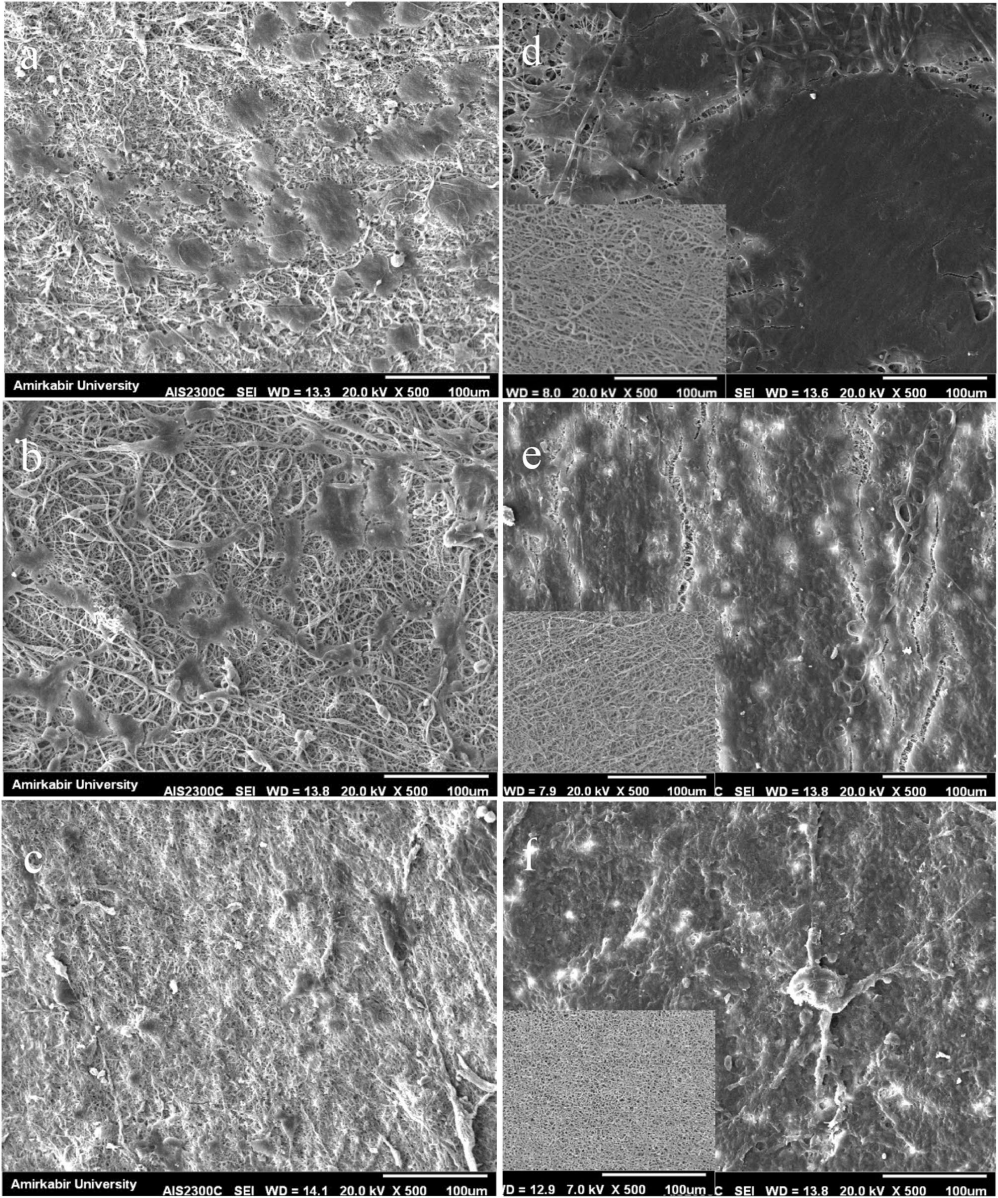
Day 1 (**a-c**) and day 3 (**d-f**) HUVECs culture on the surface of (**a**, **d**) TPU10, (**b**, **e**) TPU-SF, (**c**, **f**) TPU-SF-Hep. The SEM images of the original fiber surface are provided for comparison.

SEM images of adhered HUVECs (Fig. 8) showed that endothelial cells were attached to the surface and flattened on fibers within the first day. After the third day, cell coverage increased, and HUVECs were spread on the surface. As the figure illustrates, SF fibers enhanced the biocompatibility of electrospun fibers, and consequently, advanced the cell viability and attachment. Also, HUVECs successfully proliferated on both heparinized and non-heparinized TPU-SF scaffolds and formed a single layer of cells after the third day, which is crucial to improve the vascular graft patency rate.

#### Platelet adhesion

**Figure 9:**
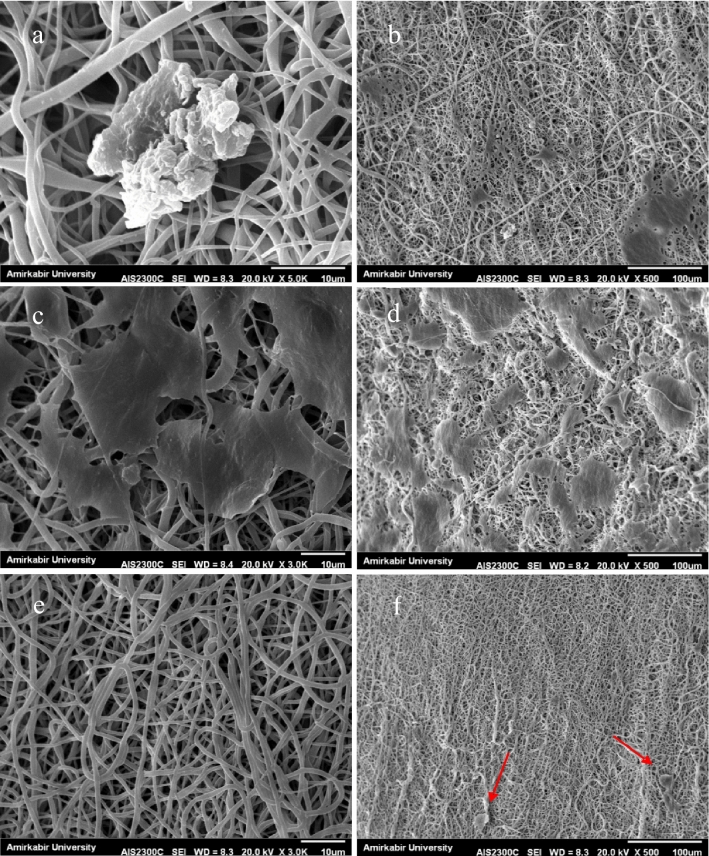
Platelet adhesion results of (**a**, **b**) TPU10 (**c**, **d**) TPU-SF and (**e**, **f**) TPU-SF-Hep (red arrows point to round-shape platelets).

A major problem of small-diameter TEVGs is rapid graft failure caused by early thrombus formation. SEM images of platelet attachment on the electrospun TPU and co-electrospun TPU-SF fibers with and without heparin showed the anti-thrombotic property of the TPU layer and the effect of heparin on the anti-thrombogenic activity of scaffolds. As illustrated in Fig. 9, large numbers of platelets attached to the surface of TPU and TPU-SF fibers and even formed clusters on the surface. On the other hand, the addition of heparin to the structure significantly decreased the number of adhered platelets on the surface of TPU-SF-Hep fibers which improves the patency compared with the absence of heparin in the structure. Therefore, based on the platelet adhesion tests, the introduction of heparin to the TPU-SF layer makes it a possible candidate for being used in vascular tissue engineering applications.

## Conclusion

In our previous studies, different types of scaffolds were implemented for regenerating tissues^8,10,58–61^. In this study, a novel scaffold was fabricated for developing small-diameter TEVGs. The growing demand for small-diameter TEVGs is the driving force behind many studies. However, several issues such as low hemocompatibility, lack of long-term patency rate, and poor biomechanical properties have not yet been addressed. An ideal TEVG should have the ability to mimic the mechanical properties of native tissues and good biocompatibility at the interface between material and the blood flow. While natural materials like SF have low immunogenicity and good biocompatibility, synthetic materials such as TPU can provide strong mechanical properties in terms of flexibility, burst pressure, and suture retention strength. Also, synthetic materials are thrombogenic and need further manipulation to reach a thromboresistant lumen. Co-electrospinning is a beneficial technique to combine the advantages of natural and synthetic biomaterials. In the present study, utilizing our previous approach, we designed, fabricated, and characterized a bilayer tubular vascular graft made up of co-electrospun TPU-SF-Hep fibers and freeze-dried C/G (2/1 v/v) hydrogel. Incorporating heparin and SF to the TPU10 fibers contributed to enhanced biocompatibility, increased wettability, and decreased platelet adhesion. Compared with previous works, the present vascular graft offered suitable mechanical properties including burst pressure, suture retention force, and tensile strength. Also, the employed method provided a non-thrombotic inner surface which was endothelialized within the first days and acted as a barrier for the migration of SMCs toward the lumen. Moreover, the highly porous outer layer supported the SMCs viability and proliferation. Thus, the as-prepared scaffold may be clinically applicable as a small diameter artificial vascular graft.
